# Global Transcriptome Sequencing Identifies Chlamydospore Specific Markers in *Candida albicans* and *Candida dubliniensis*

**DOI:** 10.1371/journal.pone.0061940

**Published:** 2013-04-15

**Authors:** Katja Palige, Jörg Linde, Ronny Martin, Bettina Böttcher, Francesco Citiulo, Derek J. Sullivan, Johann Weber, Claudia Staib, Steffen Rupp, Bernhard Hube, Joachim Morschhäuser, Peter Staib

**Affiliations:** 1 Leibniz Institute for Natural Product Research and Infection Biology – Hans Knoell Institute, Junior Research Group Fundamental Molecular Biology of Pathogenic Fungi, Jena, Germany; 2 Leibniz Institute for Natural Product Research and Infection Biology – Hans Knoell Institute, Systems Biology/Bioinformatics, Jena, Germany; 3 Center for Innovation Competence Septomics, Research Group Fungal Septomics at the Leibniz Institute for Natural Product Research and Infection Biology – Hans Knoell Institute, Jena, Germany; 4 Leibniz Institute for Natural Product Research and Infection Biology – Hans Knoell Institute, Molecular Pathogenicity Mechanisms, Jena, Germany; 5 School of Dental Science and Dublin Dental University Hospital, Trinity College Dublin, University of Dublin, Dublin, Ireland; 6 Lausanne Genomic Technologies Facility, Center for Integrative Genomics, University of Lausanne, Lausanne, Switzerland; 7 Department of Obstetrics and Gynecology, University of Würzburg, Würzburg, Germany; 8 Fraunhofer Institute for Interfacial Engineering and Biotechnology, Stuttgart, Germany; 9 Friedrich Schiller University, Jena, Germany; 10 Center for Sepsis Control and Care, Jena University Hospital, Jena, Germany; 11 Institute for Molecular Infection Biology, University of Würzburg, Würzburg, Germany; 12 Department of Research and Development, Kneipp-Werke, Würzburg, Germany; New Jersey Medical School, University of Medicine and Dentistry of New Jersey, United States of America

## Abstract

*Candida albicans* and *Candida dubliniensis* are pathogenic fungi that are highly related but differ in virulence and in some phenotypic traits. During *in vitro* growth on certain nutrient-poor media, *C. albicans* and *C. dubliniensis* are the only yeast species which are able to produce chlamydospores, large thick-walled cells of unknown function. Interestingly, only *C. dubliniensis* forms pseudohyphae with abundant chlamydospores when grown on Staib medium, while *C. albicans* grows exclusively as a budding yeast. In order to further our understanding of chlamydospore development and assembly, we compared the global transcriptional profile of both species during growth in liquid Staib medium by RNA sequencing. We also included a *C. albicans* mutant in our study which lacks the morphogenetic transcriptional repressor Nrg1. This strain, which is characterized by its constitutive pseudohyphal growth, specifically produces masses of chlamydospores in Staib medium, similar to *C. dubliniensis*. This comparative approach identified a set of putatively chlamydospore-related genes. Two of the homologous *C. albicans* and *C. dubliniensis* genes (*CSP1* and *CSP2*) which were most strongly upregulated during chlamydospore development were analysed in more detail. By use of the green fluorescent protein as a reporter, the encoded putative cell wall related proteins were found to exclusively localize to *C. albicans* and *C. dubliniensis* chlamydospores. Our findings uncover the first chlamydospore specific markers in *Candida* species and provide novel insights in the complex morphogenetic development of these important fungal pathogens.

## Introduction

The pathogenic yeast *Candida albicans* represents the clinically most important member of the genus *Candida* [1]. Although *C. albicans* is a harmless member of the normal microflora in healthy people, the species can cause life-threatening, disseminated infections in immunocompromised patients [2]. In medical routine diagnosis, *C. albicans* has been differentiated for a long time from other yeast-like fungi by a species-specific, morphogenetic characteristic, i.e. the formation of chlamydospores. These large, thick-walled, spherical cells are produced by *C. albicans* on specific nutrient-poor media such as rice-extract or corn meal agar at room temperature, typically from suspensor cells at the end of pseudohyphae [2], [3], [4], [5], [6]. Despite the importance of chlamydospores for species identification, even today, the biological function of these entities remains enigmatic [5], [7]. No role for chlamydospores has yet been identified in the life cycle of the microorganism or in fungal survival in the environment or in pathogenicity. Although readily inducible *in vitro*, chlamydospores have only rarely been observed *in vivo* [8], [9]. Chlamydospore formation would appear to be a complex process that undoubtedly requires specific genes and regulatory pathways that have been retained since the divergence of *C. albicans* and *C. dubliniensis* approximately 20 million years ago. The question therefore remains why have these species retained the capacity to produce these complex and unusual structures and what exactly is their purpose in the *Candida* life cycle?

In contrast to hyphae formation, the program of chlamydospore development has only been studied poorly at the molecular level (for review see [5]). Some of the signaling pathways which control hyphae formation in *C. albicans* were also found to influence chlamydospore development, e.g. those involving transcriptional regulators Efg1 and Nrg1, or the stress-activated protein kinase Hog1 [10], [11], [12]. Other genes, which are likely implicated in chlamydospore formation have also been identified, for example by screening libraries of deletion mutants or by testing individual knock-out strains for their ability to efficiently develop these morphological structures [13], [14]. However, so far no proteins have been identified which are specifically localized to chlamydospores. Such markers would be useful in the study of the morphogenetic development of these cellular entities, and would facilitate the differentiation of chlamydospores from other morphological growth forms.

Molecular analysis of chlamydospores has received increasing attention since the description of a new *Candida* species in 1995, *C. dubliniensis*. *C. dubliniensis* is closely related to *C. albicans* and displays many phenotypic characteristics that were assumed to be specific for *C. albicans*, including the ability to form true hyphae and chlamydospores [15], [16], [17]. Intriguingly, however, only *C. dubliniensis* was found to produce pseudohyphae and chlamydospores on Staib agar (syn. *Guizotia abyssinica* creatinine agar), where *C. albicans* grows as a budding yeast [18]. This species-specific characteristic was shown to be governed by a differential expression of the gene encoding the hyphal repressor Nrg1 in the two species. A *C. albicans* knock-out mutant in the *NRG1* gene, which is known for its constitutive pseudohyphal growth [19], [20], produces chlamydospores specifically on Staib agar, similar to *C. dubliniensis* [12].

In the present study, we set out to identify chlamydospore specific markers in *Candida*. As a method, we investigated for the first time genome wide expression patterns in *Candida* species during chlamydospore development. In detail, global transcriptomes of *C. albicans* and *C. dubliniensis* wild-type strains as well as the *C. albicans nrg1*Δ mutant were monitored by RNA sequencing during growth in Staib medium. The comparison of the detected profiles allowed the identification of a set of highly expressed genes specifically related to chlamydospore development. For selected candidates, which code for putative cell wall proteins, the chlamydospore specific expression and the exclusive localization of the encoded proteins to chlamydospores was demonstrated by green fluorescent protein (GFP) fusion strains.

## Materials and Methods

### Strains and growth conditions

*C. albicans* and *C. dubliniensis* strains used in this work are listed in Table 1. Strains were routinely propagated on YPD agar (20 g peptone, 10 g yeast extract, 20 g glucose, 15 g agar per litre) at 30°C and stored as frozen stocks in liquid YPD medium with 15% (v/v) glycerol at –80°C. Chlamydospore formation in *C. albicans* and *C. dubliniensis* was induced by growth of the strains on rice-extract agar (Beckton, Dickinson and Company, Sparks, USA) at 25°C. Staib liquid medium was used for the specific induction of chlamydospore formation in *C. dubliniensis* and the *C. albicans nrg1*Δ mutant strain MMC3 at 25°C. Staib medium was prepared like Staib agar (syn. *Guizotia abyssinica* creatinine agar) as described previously [21], [22], only the agar was omitted. In brief, 50 g pulverized *Guizotia abyssinica* plant seeds were boiled in 1 l of distilled water for 30 min, filtered and filled up to 1 l with water. Thereafter, 1 g glucose, 1 g KH_2_PO_4_ and 1 g creatinine were added before autoclaving for 20 min at 110°C.

**Table 1:** *C. albicans* and *C. dubliniensis* strains used in this study.

Candida strain	Parent	Genotype^a^	Reference
SC5314		C. albicans wild-type strain	[42]
Wü284		C. dubliniensis wild-type strain	[43]
MMC3	CAI4	Canrg1Δ::hisG-CaURA3-hisG/Canrg1Δ::hisG	[20]
Ca3512G1A/B	SC5314	orf19.3512/orf19.3512-GFP-T_ACT1_	This study
Cd30750G1A/B	Wü284	CD36_30750/CD36_30750-GFP-T_ACT1_	This study
Ca4170G1A/B	SC5314	orf19.4170/orf19.4170-GFP-T_ACT1_	This study
Cd40770G1A/B	Wü284	CD36_40770/CD36_40770-GFP-T_ACT1_	This study
Cd30750M1A/B	Wü284	CD36_30750Δ::SAT1-FLIP/CD36_307500	This study
Cd30750M2A/B	Cd30750M1A/B	CD36_30750Δ::FRT/CD36_30750	This study
Cd30750M3A/B	Cd30750M2A/B	CD36_30750Δ::FRT/CD36_30750Δ::SAT1-FLIP	This study
Cd30750M4A/B	Cd30750M3A/B	CD36_30750Δ::FRT/CD36_30750Δ::FRT	This study
Cd40770M1A/B	Wü284	CD36_40770Δ::SAT1-FLIP/CD36_40770	This study
Cd40770M2A/B	Cd40770M1A/B	CD36_40770Δ::FRT/CD36_40770	This study
Cd40770M3A/B	Cd40770M2A/B	CD36_40770Δ::FRT/CD36_40770Δ::SAT1-FLIP	This study
Cd40770M4A/B	Cd40770M3A/B	CD36_40770Δ::FRT/CD36_40770Δ::FRT	This study

a *SAT1-FLIP* denotes the SAT1 flipper cassette.

### Plasmid constructions

A DNA construct for the *CD36_30750*-*GFP* reporter fusion was generated as follows: upstream sequences plus the coding region of gene *CD36_30750* were amplified by PCR with primers Cd30750-1 and Cd30750-5, using genomic DNA from *C. dubliniensis* Wü284 as a template (all primers are listed in Table S1). Primer Cd30750-5 contains a BamHI-site which replaces the *CD36_30750* stop codon. The *GFP* gene lacking the start codon was cloned together with the *C. albicans ACT1T*-terminator by use of primers GFP1 and CaACT1T-1 and the *GFP-ACT1T* containing plasmid pSSU1G2 (unpublished data) as a template, resulting in pJetGFPACT1T1. The ApaI-BamHI *CD36_30750* fragment was cloned together with a BamHI-NcoI *GFP* fragment from pJetGFPACT1T1 in the ApaI/NcoI digested vector pSSU1G2, resulting in pCd30750G1. Finally, the downstream *SSU1* fragment in pCd30750G1 was replaced by a PstI-SacI *CD36_30750* downstream fragment obtained by PCR with primers Cd30750-6 and Cd30750-4. The resulting plasmid pCd30750G2 contains a DNA cassette which encodes *CD36_30750* which is fused at its last amino acid via Gly-Ser to the GFP (Figure S1). In the same way, GFP reporter fusions were constructed for the *C. dubliniensis* gene *CD36_40770* by use of primer pairs Cd40770-1/Cd40770-5 and Cd40770-6/Cd40770-4 and *C. albicans* genes *orf19.3512* (primer pairs 3512-1/3512-2 and 3512-3/3512-4) and *orf19.4170* (primer pairs 4170-1/4170-2 and 4170-3/4170-4), resulting in plasmids pCd40770G2, p3512G2 and p4170G2. A DNA cassette for the deletion of *CD36_30750* was constructed as follows: An ApaI-XhoI fragment with *CD36_30750* upstream sequences was cloned after PCR with the primers Cd30750-1 and Cd30750-2, using genomic DNA from *C. dubliniensis* Wü284 as a template. A SacII-SacI fragment with *CD36_30750* downstream sequences was obtained by PCR with the primers Cd30750-3 and Cd30750-4. The *CD36_30750* upstream and downstream fragments were successively cloned in order to flank the SAT1-flipper cassette as described before [23]. In the same way, a DNA cassette for the deletion of *C. dubliniensis* gene *CD36_40770* was constructed, using primer pairs Cd40770-1/Cd40770-2 and Cd40770-3/Cd40770-4, respectively.

### *C. albicans* and *C. dubliniensis* transformant construction

*C. albicans* and *C. dubliniensis* were transformed by an electroporation protocol [24] with gel-purified, linear DNA fragments from the generated plasmids: the ApaI-SacI fragments from pCd30750G2, pCd40770G2, p3512G2 and p4170G2 for integration of the GFP reporter fusions into one of the native alleles of the corresponding genes in the wild-type strains *C. dubliniensis* Wü284 and *C. albicans* SC5314, respectively (Figure S1). The ApaI-SacI fragments from pCd30750M2 and pCd40770M2 were used to delete genes *CD36_30750* and *CD36_40770*, respectively, in *C. dubliniensis* Wü284 (Figure S2). Transformants were selected on nourseothricin (Werner Bioagents, Jena, Germany), and recycling of the selection marker by the SAT1-flipping method was carried out as described before [23]. The correct insertion of the constructs was confirmed by Southern analysis.

### Southern analysis

Genomic DNA from *C. albicans* and *C. dubliniensis* was isolated as described previously [25]. A 10 µg sample of DNA was digested with appropriate restriction enzymes and separated on a 1% (w/v) agarose gel. After ethidium bromide staining, DNA was transferred by vacuum blotting onto a nylon membrane and fixed by UV cross-linking. Southern hybridization with enhanced chemiluminescence-labelled probes was performed with the Amersham ECL Direct Nucleic Acid Labelling and Detection System (GE Healthcare, Braunschweig, Germany) according to the instructions of the manufacturer.

### RNA isolation and sequencing

Total RNA from *C. albicans* and *C. dubliniensis* was isolated by the hot acidic phenol method [26], purified by use of the RNeasy Mini Kit (Qiagen, Hilden, Germany) and DNase-treated on-column with the RNase-free DNase Set (Qiagen) for removing contaminations with genomic DNA. The integrity of total RNAs was analyzed on an Agilent Bioanalyzer by monitoring the RNA integrity number (RIN). Two µg of total RNA were used to extract polyadenylated RNA and to construct strand specific RNAseq libraries according to the Illumina protocol ‘Directional mRNA-Seq Sample Preparation’ (Part # 15018460 Rev. A) (Illumina, San Diego, USA). Briefly, polyadenylated RNA was enriched by two rounds of polyA selection with oligo-dT magnetic beads. The RNA was then chemically fragmented, treated with Antarctic phosphatase (NEB) and subsequently with polynucleotide kinase (NEB). V1.5 sRNA 3′Adapter was ligated to the RNA with T4 RNA ligase2 truncated (NEB), and SRA 5′ Adapter was ligated to the RNA with T4 RNA Ligase. After ligation of the SRA RT primer, the RNA was reverse transcribed with SuperScript II Reverse Transcriptase (Invitrogen). Double stranded sequencing library DNA was then produced by 12 cycles PCR with primers GX1 and GX2. DNA was purified with AMPure XP beads (Beckman Coulter International S.A.). Library quality was validated with a Bioanalyzer 2100. Each sample was sequenced for 80 cycles on one lane of the Illumina Genome Analyzer IIx platform according to the manufacturers specifications. Yields per sample were 36 to 38 Mio pass filter reads (2.9 to 3.0 Gb).

### RNA-seq data-processing

In order to map raw sequence reads to the respective genomes, we applied the Bowtie algorithm (version 0.12.7) [27]. For all three datasets >70% of reads mapped. We applied MAID filtering [28] in order to identify differentially expressed genes. Instead of a constant (log) fold-change cutoff, MAID filtering applies a MA-plot-based signal intensity-dependent fold-change criterion. The advantage is that genes which are lowly expressed in both datasets are not defined to be differentially expressed. Due to the absence of biological replicates, we relied on the experience that the variance is higher for genes expressed at low level. To find genes which are differentially expressed between *C. dubliniensis* and *C. albicans*, we used the definition of orthologous pairs given by the Candida Genome Database (http://www.candidagenome.org/).

### Quantitative real-time (q)RT-PCR

One hundred ng of total RNA were used to perform qRT-PCR with a one step approach using the Brilliant III SYBR Green Ultra-Fast QRT PCR master mix kit (Agilent Technologies, La Jolla, USA). RT-PCR was performed on a Stratagene Mx3005P and the threshold cycle was determined by the instrument’s MxPro software version 4.10 (Agilent Technologies, La Jolla, USA). By the ΔΔCT method [29] expression was calculated and normalized to the expression of the *CaACT1/CdACT1* gene. For all samples, three biological replicates were analyzed. Data were expressed as the mean ± SD. Differences were analyzed by the two-tailed unpaired Student’s *t*-test, a *P* value of <0.05 was considered statistically significant.

### Analysis of DNA/protein sequence identity and similarity

Pairwise sequence alignments were conducted by use of the free available Needleman-Wunsch global alignment tool (Needle) at *The European Molecular Biology Open Software Suite* (emboss, http://emboss.open-bio.org/).

### Fluorescence microscopy

Fluorescence microscopy was performed with a Zeiss Axio-Observer Z1 microscope equipped with a Zeiss HXP120C illuminator. Images were acquired by use of the corresponding filter settings for green fluorescent protein (GFP) and parallel/overlay transmission images. The cells were inspected with a x40 objective. Surface plot analysis to localize the fluorescence signal of CdCsp1/2-GFP was performed with ImageJ 1.46r.

## Results and Discussion

### *C. dubliniensis* wild type and the *C. albicans nrg1*Δ mutant form chlamydospores during growth in Staib liquid medium

**Figure 1:**
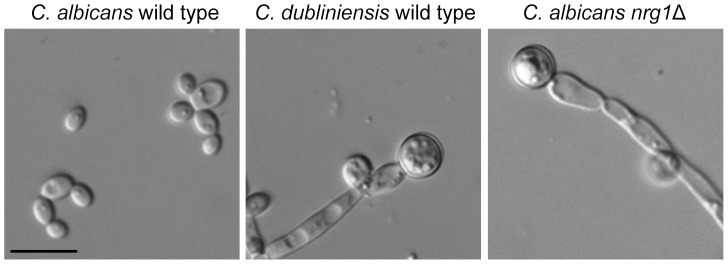
Differential chlamydospore development by the analyzed *Candida* strains in Staib liquid medium. *C. dubliniensis* wild type Wü284 and the *C. albicans nrg1*Δ mutant MMC3 form chlamydospores, in contrast to the *C. albicans* wild type SC5314. The fungal strains were grown for 28 h in Staib medium at 25°C and inspected by microscopy (scale bar: 10 µm).

As previously reported, the *C. dubliniensis* wild type and the *C. albicans nrg1*Δ mutant produce chlamydospores during growth on Staib agar, in contrast to the *C. albicans* wild type [12]. First, we proved whether a similar, expected growth phenotype of the three analyzed strains is also displayed in Staib liquid medium [30], since liquid culture conditions facilitated the planned transcriptome analysis. We found an incubation for 28 h at 25°C optimal for chlamydospore analysis in Staib liquid medium. At this time point, both the *C. dubliniensis* wild type as well as the *C. albicans nrg1*Δ mutant exclusively grew in form of pseudohyphae, almost all of which produced chlamydospores at their terminal ends (Figure 1). It has to be noted that the *C. albicans nrg1*Δ mutant constitutively forms pseudohyphae, but not chlamydospores. Instead, the formation of chlamydospores by *C. albicans nrg1*Δ pseudohyphae is specifically induced in Staib medium, hence allowing the identification of putative chlamydospore related genes by comparative gene expression analysis.

### Comparative RNA sequencing identifies putative chlamydospore specific genes

**Figure 2:**
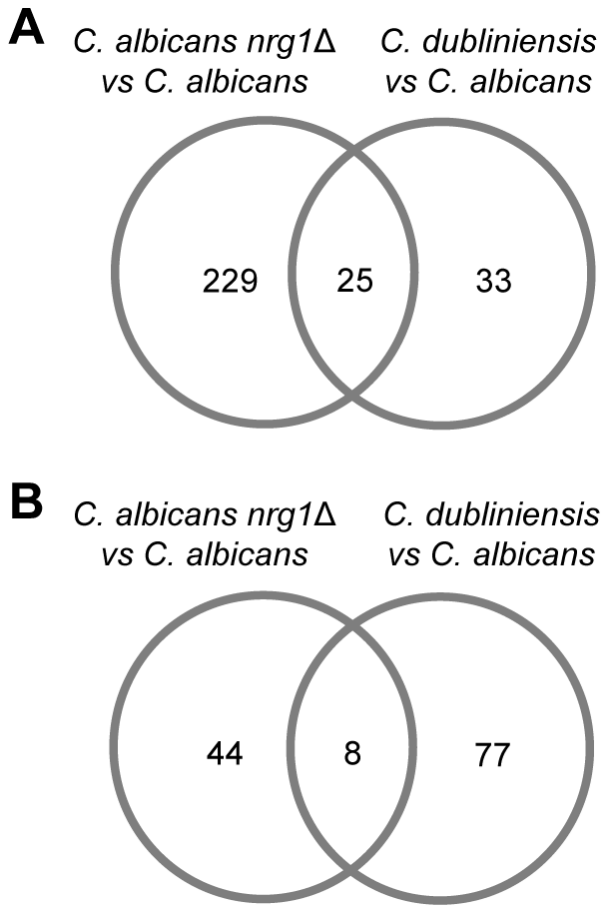
Identification of chlamydospore specific genes in *Candida*. Venn diagram of genes which were ≥ two fold up- (A) and downregulated (B) in both the *C. albicans nrg1*Δ mutant and the *C. dubliniensis* wild type during growth in Staib medium.

Total RNA from the three tested *Candida* strains, i.e. *C. albicans* wild type SC5314, *C. albicans* MMC3 (*nrg1*Δ) and *C. dubliniensis* wild type Wü284, was isolated after 28 h of growth in Staib liquid medium and used for global RNA sequence analysis (Materials and Methods). The complete results of pairwise relative gene expression comparisons of the three strains is depicted in Table S2. In order to identify differentially regulated genes, we applied the stringent MAID filtering approach (MA-plot-based signal intensity-dependent fold-change) [28], permitting the removal of genes which are expressed at a low level in both compared conditions/strains. A set of putative chlamydospore formation related genes was obtained by comparing datasets from the *C. albicans nrg1*Δ mutant strain and the *C. dubliniensis* wild type with the *C. albicans* wild type. By this approach, we identified 25 strongly up- and 8 downregulated genes, respectively (Figure 2, Table 2), most of which were uncharacterized. Since chlamydospore related gene expression has not been monitored before on a global scale, the identification of many unknown function/uncharacterized genes points to the assumed specificity of the chlamydospore developmental program. Interestingly, many of the highly upregulated genes encode putative cell wall/plasma membrane associated proteins, including *PGA13* and *PGA55* [31], [32]. This finding suggests that chlamydospore cell walls contain a characteristic composition of proteins. Based on our findings specified further on in this work we designated the two highly upregulated genes *orf19.3512/CD36_30750* and *orf19.4170/CD36_40770* as *C. albicans* and *C. dubliniensis* ‘*C*hlamydospore *S*pecific *P*rotein *1* and *2*′, i.e. *ca/cdCSP1* and *ca/cdCSP2*, respectively (Table 2).

**Table 2:** Strongly differentially expressed genes in *C. albicans nrg1*Δ and *C. dubliniensis* vs. *C. albicans* in Staib medium.

orf19 ID	Name^a^	Dub ID	Relative Expression (log2fold)^b^	
			***C. albicans nrg1*** **Δ vs. ** ***C. albicans***	***C. dubliniensis*** ** vs. ** ***C. albicans***
*orf19.3512*	*CSP1*	*CD36_30750*	9.60	17.20
*orf19.654*		*CD36_30570*	9.50	17.90
*orf19.4170*	*CSP2*	*CD36_40770*	8.47	12.31
*orf19.4463*		*CD36_03620*	8.38	13.47
*orf19.2317*		*CD36_10300*	7.13	9.93
*orf19.6315*		*CD36_30140*	6.49	8.29
*orf19.6420*	*PGA13*	*CD36_34100*	6.47	6.93
*orf19.2638*		*CD36_53200*	6.44	9.25
*orf19.4011*		*CD36_54780*	6.20	10.82
*orf19.2583*		*CD36_26850*	5.61	5.83
*orf19.6741*		*CD36_87380*	5.35	8.82
*orf19.207*	*PGA55*	*CD36_23160*	4.84	6.93
*orf19.3330.3*	*POX18*	*CD36_01320*	4.34	7.04
*orf19.3885*		*CD36_31810*	3.93	7.48
*orf19.4688*	*DAG7*	*CD36_41020*	3.70	4.58
*orf19.2457*		*CD36_05580*	3,44	4.83
*orf19.4783*		*CD36_08720*	2.96	5.74
*orf19.6569*		*CD36_71400*	2.84	5.21
*orf19.4264*		*CD36_52290*	2.84	5.03
*orf19.4459*		*CD36_03600*	2.74	5.16
*orf19.6920*		*CD36_71220*	2.64	4.82
*orf19.2506*		*CD36_80940*	2.48	4.55
*orf19.4953*		*CD36_12310*	2.36	4.94
*orf19.6788*		*CD36_87050*	2.07	4.49
*orf19.5645*	*MET15*	*CD36_40270*	1.92	5.28
*orf19.508*	*QDR1*	*CD36_29520*	–6.43	–6.32
*orf19.4773*	*AOX2*	*CD36_08630*	–6.03	–6.19
*orf19.7150*	*NRG1*	*CD36_73890*	–4.37	–4.98
*orf19.7554*		*CD36_34960*	–3.21	–5.31
*orf19.1189*		*CD36_60240*	–2.49	–5.49
*orf19.2251*	*AAH1*	*CD36_21260*	–1.78	–4.55
*orf19.1193*	*GNP1*	*CD36_60280*	–1.75	–4.99
*orf19.4555*	*ALS4*	*CD36_64610*	–1.63	–10.47

a Denominations *CSP1* and *CSP2* were proposed in the present study.

b Downregulation of genes is indicated by a minus (–), followed by the logarithmised log fold change value.

The hyphal repressor Nrg1 was previously found to govern the differential chlamydospore phenotype of *C. albicans* and *C. dubliniensis* in Staib medium [12]. Therefore, it appears reasonable that some of the detected genes were previously related to filamentation and/or annotated as putative targets of Nrg1. Examples include *PGA13*, *PGA55* and *IHD1*, which encode putative glycosylphosphatidylinositol (GPI)-anchored proteins [31], the latter being named as ‘induced during hyphae development’ [20], [33]. Other candidate target genes are the strongly upregulated unknown function gene *orf19.6741* as well as *UME6*, which is described as a filament specific regulator in *C. albicans* (Table 2; Table S2) [34], [35]. Notably, it has been reported that a differential expression of the *UME6* gene contributes to the varied ability of *C. albicans* and *C. dubliniensis* to form filaments [36]. Confirming prior results on *NRG1* expression during chlamydospore development [12], we found the gene to be strongly downregulated in *C. dubliniensis* versus the *C. albicans* wild-type strain during growth in Staib medium (Table 2, Table S2).

Analysis of RNAseq data did not detect a particular differential induction of genes that have previously been identified by mutant screening to be important for efficient chlamydospore formation in *C. albicans*, such as *ISW2*, *MDS3*, *RIM13*, *RIM101*, *SCH9* and *SUV3* [14]. Supporting former findings on the role of the stress-activated protein kinase Hog1 for chlamydospore production in *C. albicans*, *HOG1* transcription was detected in our experiments to be elevated in *C. dubliniensis* and the *C. albicans nrg1*Δ mutant, suggesting that the cells grown under these conditions may be experiencing osmotic or nutritional stress.

Other genes of interest which were strongly upregulated during chlamydospore development in our assay were those which by differential expression patterns were previously detected during switching or mating in *C. albicans*. For example, expression of *orf19.2317* and *DAG7* was shown to be inducible by alpha-pheromone [37], and *orf19.2506* was reported to be opaque cell specific [38]. In addition, one of the most strongly downregulated genes associated with chlamydospore development was *QDR1*/*CD36_29520*. This gene, which encodes a putative transporter, was previously detected in *C. albicans* to also experience strong differential expression in white versus opaque cells [39]. The discovery of mating in *C. albicans* has revealed that the life cycle of this microorganism is more complex than originally assumed. In this context, the interest in chlamydospore development should also be restimulated.

### Sequence specificity of the four genes which were most strongly upregulated during chlamydospore formation

Intriguingly, the four most strongly upregulated *C. albicans* genes and *C. dubliniensis* homologues, repectively, encode putative cell wall proteins which display considerable similarity (Table 3) (http://old.genedb.org/genedb/cdubliniensis/; http://www.candidagenome.org/). Given their chlamydospore related expression and the fact that only these two *Candida* species form chlamydospores, we asked whether these genes are specific for *C. dubliniensis* and *C. albicans* in the genus *Candida*. The Candida Gene Order Browser (GCOB) is an online tool for visualising the syntenic context of genes from multiple *Candida* genomes (http://cgob.ucd.ie; [40]). Among 14 species included in GCOB, *caCSP1* (*orf19.3512*) and *orf19.654* related genes were only found in *C. albicans* and *C. dubliniensis*. In case of *C. albicans caCSP2* (*orf19.4170*), a homologue was in addition to *C. dubliniensis* also detected in *C. tropicalis* (*CTRG_01767*). A comparison of *C. albicans caCSP2* and *C. tropicalis CTRG_01767* on the level of the deduced proteins revealed identity/similarity of 38.9/47.7%, whereas the proteins encoded by *caCSP2* and *cdCSP2* showed identity/similarity of 83.8/86.7% (Table 3). Whether the identified *C. tropicalis* gene encodes a functional homologue of *CSP2* is questionable, especially since *C. tropicalis* is not known to produce chlamydospores. In the case of *orf19.4463*, which was absent from the other inspected *Candida* species, the homologous gene *CD36_03620* in *C. dubliniensis* is annotated as a pseudogene which contains several stop codons. The deduced proteins encoded by *orf19.4463* and *CD36_03620* displayed identity/similarity of 30.6/38.3% (Table 3), but the genes showed 59.6% identity on the level of DNA. Overall, the application of GCOB uncovered that the identified, putative chlamydospore related *C. albicans* and *C. dubliniensis* genes are not widely distributed in the genus *Candida*. This observation further underlines a putative specific role of these factors during chlamydospore development in *C. albicans* and *C. dubliniensis*.

**Table 3:** Protein sequence identity/similarity among gene products encoded by the four genes most strongly upregulated during chlamydospore formation.

C. albicans	C. dubliniensis	% identity/similarity	% identity (DNA)^a^
caCSP1	cdCSP1	80.1/83.2	78.3
orf19.654	CD36_30570	80.2/89.2	84.1
caCSP2	cdCSP2	83.8/86.7	82.0
orf19.4463	CD36_03620	30.6/38.3	59.6

	caCSP1^b^	orf19.654	caCSP2	orf19.4463
**caCSP1**		47.5	55.8	36.8
**orf19.654**	58.3		56.0	65.7
**caCSP2**	60.2	67.9		51.4
**orf19.4463**	48.2	72.2	64.4	

	cdCSP1	CD36_30570	cdCSP2	CD36_03620
**cdCSP1**		42.6	59.0	26.7
**CD36_30570**	56.6		60.4	40.5
**cdCSP2**	64.5	76.0		29.0
**CD36_03620**	38.0	47.4	37.2	

a Per cent identity of the *C. albicans* and *C. dubliniensis* homologues on the level of DNA.

b Per cent similarity (lower left) and identity (upper right) among the gene products is given.

### Expression of *ca/cdCSP1* and *ca/cdCSP2* is specifically correlated with chlamydospore development

**Figure 3:**
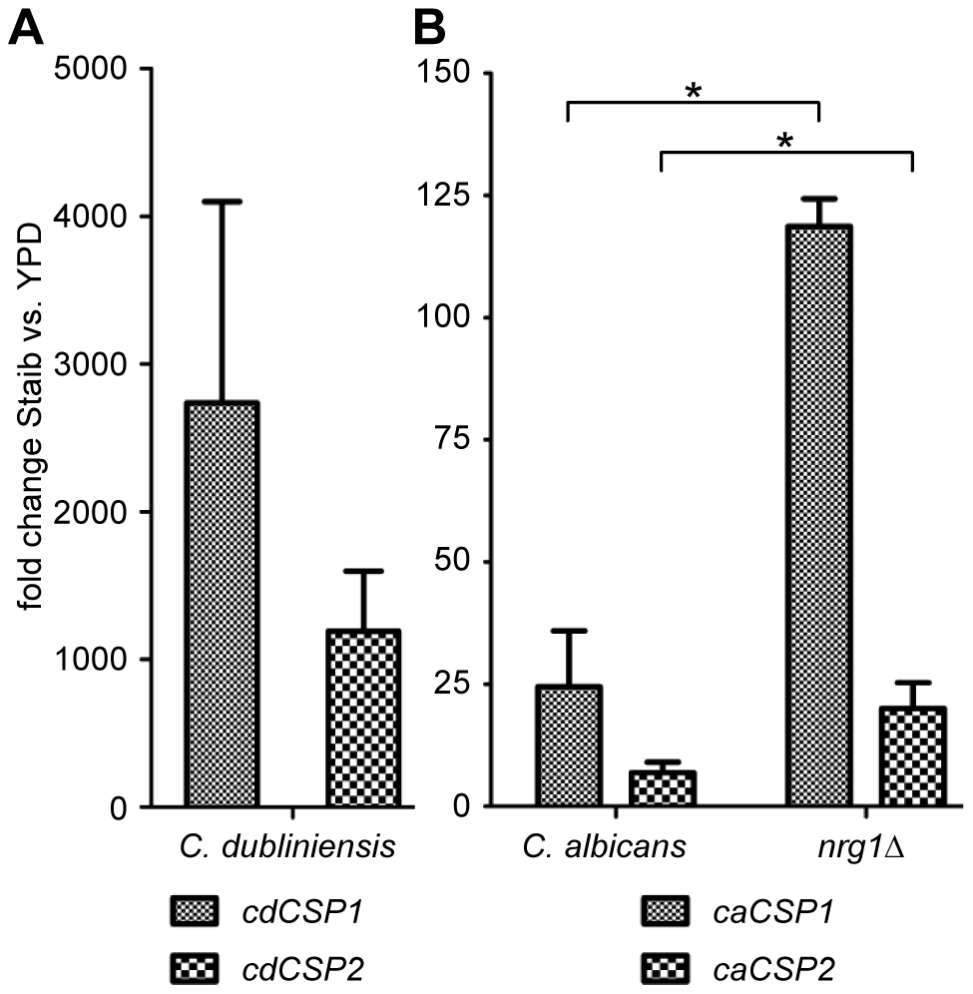
Induced expression levels of genes *CSP1* and *CSP2* during chlamydospore formation. *C. dubliniensis* Wü284, *C. albicans* SC5314 and the *C. albicans nrg1*Δ mutant were grown for 28 h in Staib medium and YPD medium, respectively, before total RNA was isolated. (A) qRT-PCR measurements detected a strong upregulation of *cdCSP1* and *cdCSP2* gene expression levels in *C. dubliniensis* during growth in Staib versus YPD medium. (B) Similarly, the *C. albicans* homologues *caCSP1* and *caCSP2* were found to be upregulated in the chlamydospore producing *C. albicans nrg1*Δ mutant stronger than in *C. albicans* wild-type yeast cells. The results are the means ±SD from three biological replicates, ‘*’ indicates that the detected differences were significant (*P*<0.05).

In search of chlamydospore specific markers two pairs of *C. albicans* and *C. dubliniensis* homologues were selected from our identified set of chlamydospore development related genes for detailed analysis, i.e. *cdCSP1*/*caCSP1* and *cdCSP2*/*caCSP2*. According to the *C. dubliniensis* genome database, these genes putatively encode cell wall associated proteins (http://old.genedb.org/genedb/cdubliniensis/). A comparison of the deduced amino acid sequences revealed that homologues *cdCSP1* and *caCSP1* display 80.1/83.2% identity/similarity. Homologues *cdCSP2* and *caCSP2* are identical/similar to 83.8/86.7% (Table 3). The expression of these highly upregulated, putative chlamydospore related *C. dubliniensis* and *C. albicans* genes was confirmed by qRT-PCR analysis (Figure 3). The results show that *cdCSP1* and *cdCSP2* were upregulated >1000-fold in *C. dubliniensis* during growth in Staib medium in comparison to growth in YPD medium. In accordance, expression levels of the *C. albicans* homologues *caCSP1* and *caCSP2* were higher in the *C. albicans nrg1*Δ mutant than in the *C. albicans* wild type during growth in Staib versus YPD medium. These observations made the two selected *C. albicans* and *C. dubliniensis* genes promising candidates for chlamydospore specific markers.

### Localization of chlamydospore specific markers in *C. dubliniensis* and *C. albicans*

**Figure 4:**
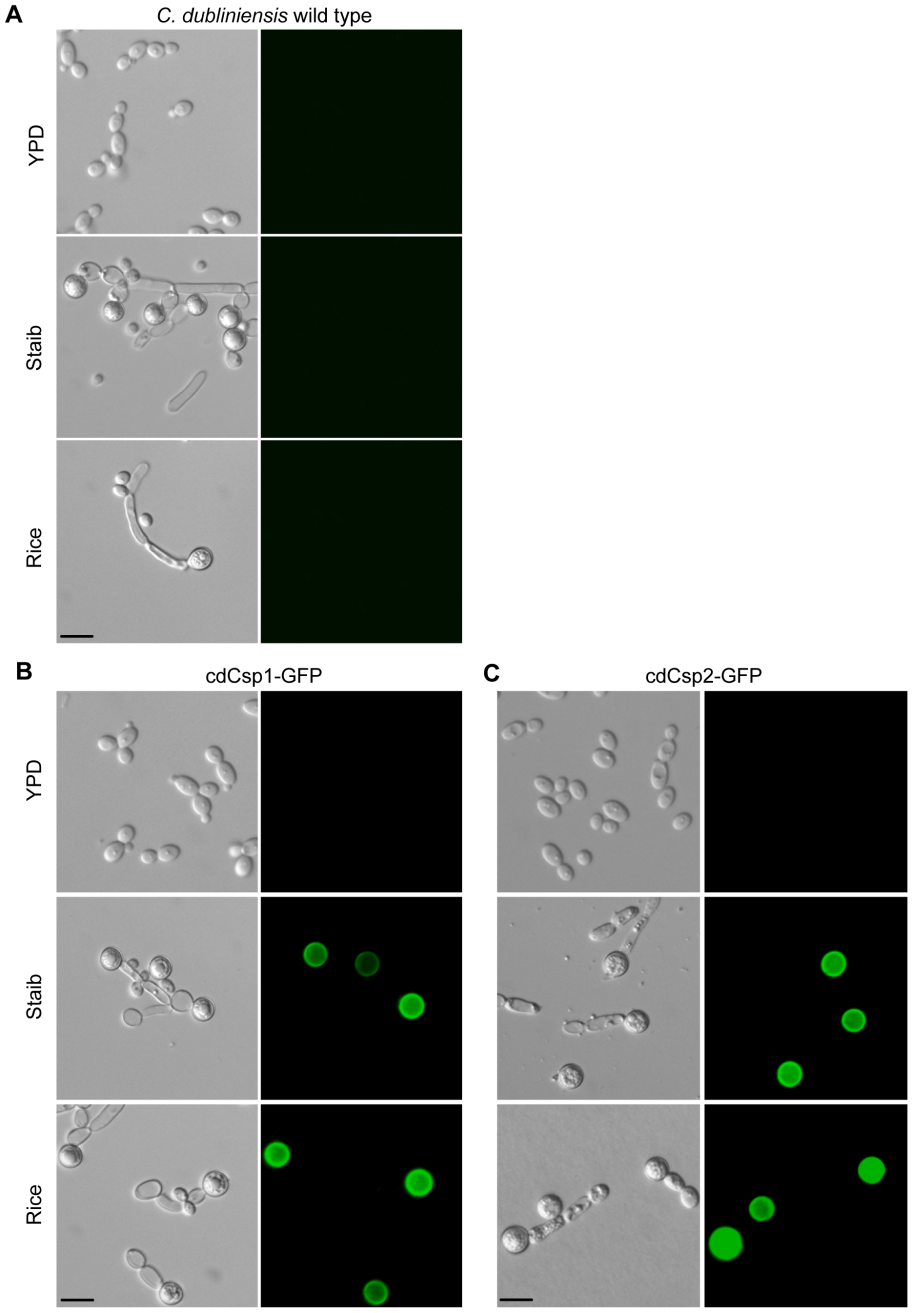
Proteins encoded by *cdCSP1* and *cdCSP2* are specifically expressed and located in chlamydospores. *C. dubliniensis* wild type Wü284 (A) and the *C. dubliniensis* GFP reporter strains Cd30750G1A/B (cdCsp1-GFP) (B) and Cd40770G1A/B (cdCsp2-GFP) (C) were grown in YPD and Staib liquid medium for 28 h at 25°C, and on rice-extract agar for 3 d at 25°C, respectively, and inspected by phase contrast and fluorescence microscopy. Fluorescence microscopy demonstrated that the genes of interest are specifically induced during growth in Staib medium and that the encoded proteins exclusively localize to chlamydospores. The two independently constructed A/B-GFP reporter strains behaved identically and only one of them is shown (scale bar: 10 µm).

The putative chlamydospore related expression of the *C. dubliniensis* genes *cdCSP1* and *cdCSP2* was next analysed in the context of morphologic development. In order to define the expression on a cellular level and the morphotype specific localization of the encoded proteins, DNA cassettes for translational fusions with the green fluorescent protein (GFP) were constructed and integrated into one of the corresponding alleles of the *C. dubliniensis* wild type Wü284 (Figure S1 and data not shown). Cells of the wild type and resulting reporter strains Cd30750G1A/B (for *cdCSP1*) and Cd40770G1A/B (for *cdCSP2*) were grown in Staib medium for 28 h and inspected by fluorescence microscopy. Growth in YPD medium was used as a control. As demonstrated in Figure 4, the analysed proteins were not only specifically and highly abundant in *C. dubliniensis* cells grown in Staib medium, but were exclusively expressed and located in chlamydospores. In these entities, fluorescence was most intensive at the cell surface, thus supporting the putative function of cdCsp1 and cdCsp2 as cell wall proteins. Localization of cdCsp1/2 to chlamydospore cell walls was further supported by a surface plot analysis, shown as example for cdCsp1 (Figure S3). Expression of these proteins was not detected in yeast cells or pseudohyphae. Most notably, fluorescence was even not detected in suspensor cells, which carry chlamydospores at their terminal ends and presumably share a continous outer layer with them [41]. In order to find out whether the identified proteins cdCsp1 and cdCsp2 are also specifically expressed in *C. dubliniensis* chlamydospores induced by conditions different from Staib medium, the reporter strains were also monitored during growth on rice-extract agar. Like in Staib medium, the investigated gene products were found to be specifically localized to chlamydospores (Figure 4). *C. dubliniensis* wild-type cells grown in YPD as well as under the tested chlamydospore inducing conditions were used as negative controls in order to exclude unspecific autofluorescence.

**Figure 5:**
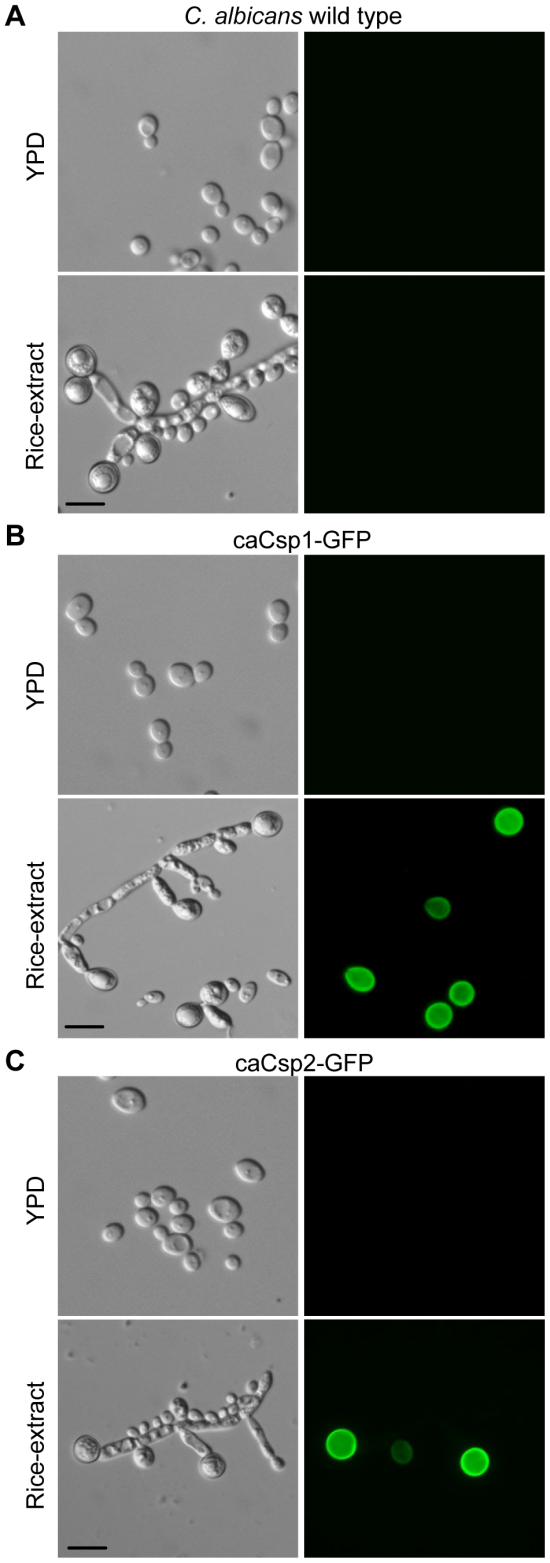
Proteins encoded by *C. albicans caCSP1* and *caCSP2* are specifically expressed and located in chlamydospores. *C. albicans* wild type SC5314 (A) and the *C. albicans* GFP reporter strains Ca3512G1A/B (caCsp1-GFP) (B) and Ca4170G1A/B (caCsp2-GFP) (C) were grown in YPD medium for 28 h at 25°C and on rice-extract agar for 3 d at 25°C, respectively, before the fungal cells were inspected by phase contrast and fluorescence microscopy. Note that the monitored proteins exclusively localize to chlamydospores. The two independently constructed A/B-GFP reporter strains behaved identically and only one of them is shown (scale bar: 10 µm).

Next, we investigated whether the identified chlamydospore related factors are also specifically expressed in *C. albicans* chlamydospores. For this purpose, GFP reporter fusions with the homologous *C. albicans* genes *caCSP1* and *caCSP2*, respectively, were constructed and integrated into the genome of the wild type SC5314 at the corresponding loci (Figure S1 and data not shown). The wild type and the resulting reporter strains Ca3512G1A/B (for *caCSP1*) and Ca4170G1A/B (for *caCSP2*) were grown on rice-extract agar and inspected by fluorescence microscopy. Like in *C. dubliniensis*, the monitored *C. albicans* proteins were also specifically expressed and localized in chlamydospores (Figure 5). This finding supported the notion that the proteins encoded by genes *ca/cdCSP1* and *ca/cdCSP2* are the first identified, strictly chlamydospore related factors in *Candida*. Moreover, these proteins appear to be useful as markers for these morphological entities, and the constructed GFP reporter may be useful tools for future research.

### Analysis of *C. dubliniensis* mutants in *cdCSP1* and *cdCSP2*

Since the function of chlamydospores is unknown, we were not able to investigate the role of the identified chlamydospore specific proteins. Nevertheless, in case of *C. dubliniensis*, we tested whether the two analysed genes *cdCSP1* and *cdCSP2* are required for efficient chlamydospore development. Deletion mutants Cd30750M4A/B (*cdcsp1*Δ) and Cd40770M4A/B (*cdcsp2*Δ) were constructed in the wild-type strain Wü284 by use of the SAT1-flipper technology (Figure S2). An altered ability of these mutants to produce chlamydospores in Staib medium was not observed (Figure S4). No difference to the wild type was also detected when these mutants were assayed for germ tube formation in cell culture medium or when growth sensitivity was tested in the presence of calcofluor white, congo red, menadion or hydrogen peroxide (data not shown). However, as indicated above (Table 3), the chlamydospore specific genes *cdCSP1*, *CD36_30570*, *cdCSP2* and *CD36_03620* display a high degree of similarity. Therefore, redundant functions of the encoded proteins during chlamydospore assembly can not be excluded, thereby masking a potential phenotype of the single knock-out mutants in either *cdCSP1* or *cdCSP2*. Future construction and phenotypic analyses of multiple knock-out strains in all these related genes would allow further insights in their potential structural functions. Moreover, mutants in the identified chlamydospore specific genes may further be investigated once a functional role of chlamydospores is known.

## Conclusion

To date, it remains obscure whether the ability to produce chlamydospores has any impact on the basic life cycle or the adaptation of *C. albicans* and *C. dubliniensis* to their human host. Interestingly, however, especially these two pathogenic *Candida* species, which are usually not found in the environment [2], [16], can form these mysterious morphological structures. In order to get novel insights into the biological role of chlamydospores, the molecular analysis of their development and structural assembly appears therefore to be of particular interest. In the present study, we addressed this issue by the identification of genes which encode chlamydospore specific factors. We took advantage of the observation that *C. albicans* and *C. dubliniensis* display a species specific difference in the regulation of chlamydospore formation in response to environmental growth conditions, i.e. by incubation in Staib medium [18]. The knowledge that species specific chlamydospore production under these conditions is controled by the differential expression of the transcriptional repressor Nrg1 further supported the identification of chlamydospore specific genes. In general, the identification of chlamydospore associated factors may be difficult, given the observation that chlamydospore production is usually correlated with pseudohyphae formation – although it is not clear whether these two phenotypes depend on each other or represent independent, co-regulated pathways. In this context, the discovered chlamydospore specific proteins, together with the provided GFP reporter constructs will further help elucidating the genetic control of chlamydospore related gene expression in *Candida*.

In general, knowledge of the identified ‘chlamydospore-specific’ markers may have particular practical value for chlamydospore identification as well as for further detailed studies on chlamydospore formation, maintenance and germination. Future studies on supposed ‘chlamydospore specific’ markers will elucidate whether such factors participate in additional processes as well, for example during host adaptation or mating. If chlamydospores played no role in the life cycle of *C. albicans* and *C. dubliniensis* one would have expected either or both species to have lost the capability to synthesise them. However, since these related pathogenic species are the only yeasts to have been observed to produce chlamydospores it remains to be seen how these fungi benefit from this phenotype. Pursuing research on chlamydospores may not only identify a role for these intriguing cells, but may also help clarify the complete life cycle of *C. albicans* and solve the riddle, why *C. albicans* has not lost the ability to form these striking cellular structures during evolution.

## Supporting Information

Figure S1 **Construction of GFP reporter strains.** (A) The structure of the insert of plasmid pCd30750G2 containing the *CD36_30750(cdCSP1)*-*GFP* reporter fusion is shown on top. At the bottom, the genomic structure of the *CD36_30750* locus in strain Wü284 is shown. The *CD36_30750* coding region is represented by the white arrow, the upstream and downstream regions by solid lines. The *GFP* gene, which is fused to the last codon (before the stop codon) of *CD36_30750*, is symbolized by the hatched arrow. The *caSAT1* selection marker is marked by a grey arrow. Probes for Southern analysis of transformants are indicated by black bars. Restriction sites used to obtain the linear fragment and for Southern analysis are: A, ApaI; SI, SalI; ScI, SacI. (B) Southern hybridization of SalI-digested genomic DNA of parental strain *C. dubliniensis* Wü284 (lane 1) and GFP reporter strains Cd30750G1A (lane 1) and Cd30750G1B (lane 2) with the *CD36_30750*-specific probe 1. The sizes of the hybridizing fragments (in kilobases) are given on the left side of the blot, their identities on the right. (C) Southern hybridization of XbaI-digested genomic DNA of parental strain *C. albicans* SC5314 (lane1) and the GFP reporter strains Ca3512G1A (lane 2) and Ca3512G1B (lane 3) with the *orf19.3512*-specific probe 1. A restriction site polymorphism allows the differentiation of the two homologous wild-type alleles. Reporter strains Ca3512G1A/B containing the *orf19.3512(caCSP1)-GFP* fusion were constructed in the same way as the *C. dubliniensis GFP-*reporter strains.
(TIF)

Figure S2 **Construction of** ***C. dubliniensis*** **knock-out mutants in** ***cdCSP1*** **(** ***CD36_30750*** **) and** ***cdCSP2*** **(** ***CD36_40770*** **), respectively.** (A) Structure of the deletion cassette from plasmid pCd30750M2 (top), which was used for deletion of both *CD36_30750* alleles, and genomic structure of the *CD36_30750* locus in strain Wü284 (bottom). The *CD36_30750* coding region is represented by the white arrow, the upstream and downstream regions by the solid lines. The SAT1 flipper cassette is represented by a grey rectangle bordered by *FRT* sites (black arrows). The 34-bp *FRT* sites are not drawn to scale. The probes which were used for Southern analysis of the transformants are indicated by the black bars. Restriction sites used to cut out the linear fragment from the plasmid and for Southern analysis are given: A, ApaI; SI, SalI; ScI, SacI. (B) Southern hybridization of SalI-digested genomic DNA of parental strain Wü284 (lane 1), heterozygous *CD36_30750*Δ mutants Cd30750M2A (lane 2) and Cd30750M2B (lane 3), homozygous *CD36_30750*Δ mutants Cd30750M4A (lane 4) and Cd30750M4B (lane 5) with the *CD36_30750*-specific probe 1. The sizes of the hybridizing fragments (in kilobases) are given on the left side of the blot, and their identities on the right. (C) Southern hybridization of EcoRV-digested genomic DNA of parental strain Wü284 (lane 1), heterozygous *CD36_40770*Δ mutants Cd40770M2A (lane 2) and Cd40770M2B (lane 3), homozygous *CD36_40770*Δ mutants Cd40770M4A (lane 4) and Cd40770M4B (lane 5) with the *CD36_40770*-specific probe 1. The sizes of the hybridizing fragments are given on the left side of the blot, and their identities on the right.
(TIF)

Figure S3 **Protein localization of cdCsp1-GFP in chlamydospores.** Fluorescence microscopy pictures of *C. dubliniensis* chlamydospores of strain Cd30750G1A after growth for 3 d at 25°C on rice-extract agar were analysed by surface plot analysis to localize the fluorescence signal of cdCsp1-GFP. (A) The yellow rectangle marks the area for the surface plot analysis. The intensity of fluorescence signal of cdCsp1-GFP within the defined region was determined by plot analysis (not shown) and surface plot analysis (B). The highest brightness was detected within the outer layer of the chlamydospore, suggesting that cdCsp1 is particularly located within the chlamydospore cell wall.
(TIF)

Figure S4 **Phenotypic analysis of** ***C. dubliniensis*** **knock-out mutants in genes** ***cdCSP1*** **and** ***cdCSP2*** **, respectively.** *C. dubliniensis* wild type Wü284 and mutant strains Cd30750M4A/B (*cdcsp1*Δ) and Cd40770M4A/B (*cdcsp2*Δ) were grown for 28 h in Staib medium, before cellular morphology was inspected by microscopy. Like the wild type control, both mutants efficiently produced chlamydospores under the tested conditions. For each mutant, the two independently constructed transformants (A/B) behaved identically and only one of them is shown.
(TIF)

Table S1 **Primers used in this study.**
(DOC)

Table S2 **Identification of genes that were differentially regulated during chlamydospore development.** The table contains the relative expression values (log2fold) for all *C. albicans* and *C. dubliniensis* genes detected in the comparative RNAseq analyses, i.e. the *C. albicans nrg1*Δ mutant versus *C. albicans* wild type, *C. dubliniensis* wild type versus *C. albicans* wild type as well as *C. dubliniensis* wild type versus the *C. albicans nrg1*Δ mutant.
(XLS)
