# Supplementary figures and images for: Global Transcriptome Sequencing Identifies Chlamydospore Specific Markers in Candida albicans and Candida dubliniensis
Source: PLoS One. 2013 Apr 15;8(4):e61940. doi: 10.1371/journal.pone.0061940 (PMC3626690; doi:10.1371/journal.pone.0061940)

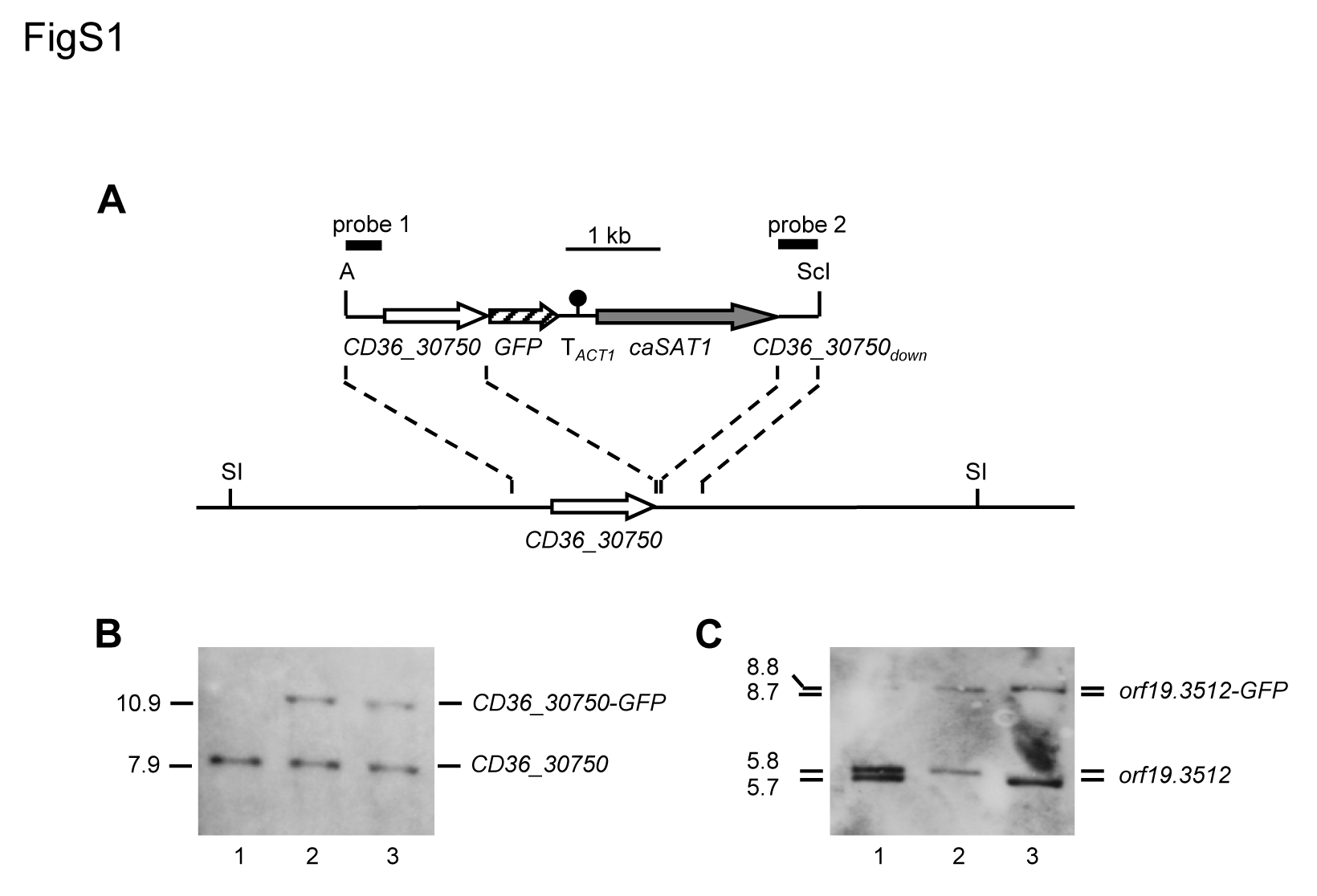

Supplement: Figure S1 — Construction of GFP reporter strains. (A) The structure of the insert of plasmid pCd30750G2 containing the CD36_30750(cdCSP1)-GFP reporter fusion is shown on top. At the bottom, the genomic structure of the CD36_30750 locus in strain Wü284 is shown. The CD36_30750 coding region is represented by the white arrow, the upstream and downstream regions by solid lines. The GFP gene, which is fused to the last codon (before the stop codon) of CD36_30750, is symbolized by the hatched arrow. The caSAT1 selection marker is marked by a grey arrow. Probes for Southern analysis of transformants are indicated by black bars. Restriction sites used to obtain the linear fragment and for Southern analysis are: A, ApaI; SI, SalI; ScI, SacI. (B) Southern hybridization of SalI-digested genomic DNA of parental strain C. dubliniensis Wü284 (lane 1) and GFP reporter strains Cd30750G1A (lane 1) and Cd30750G1B (lane 2) with the CD36_30750-specific probe 1. The sizes of the hybridizing fragments (in kilobases) are given on the left side of the blot, their identities on the right. (C) Southern hybridization of XbaI-digested genomic DNA of parental strain C. albicans SC5314 (lane1) and the GFP reporter strains Ca3512G1A (lane 2) and Ca3512G1B (lane 3) with the orf19.3512-specific probe 1. A restriction site polymorphism allows the differentiation of the two homologous wild-type alleles. Reporter strains Ca3512G1A/B containing the orf19.3512(caCSP1)-GFP fusion were constructed in the same way as the C. dubliniensis GFP-reporter strains. (TIF) [file pone.0061940.s001.tif]

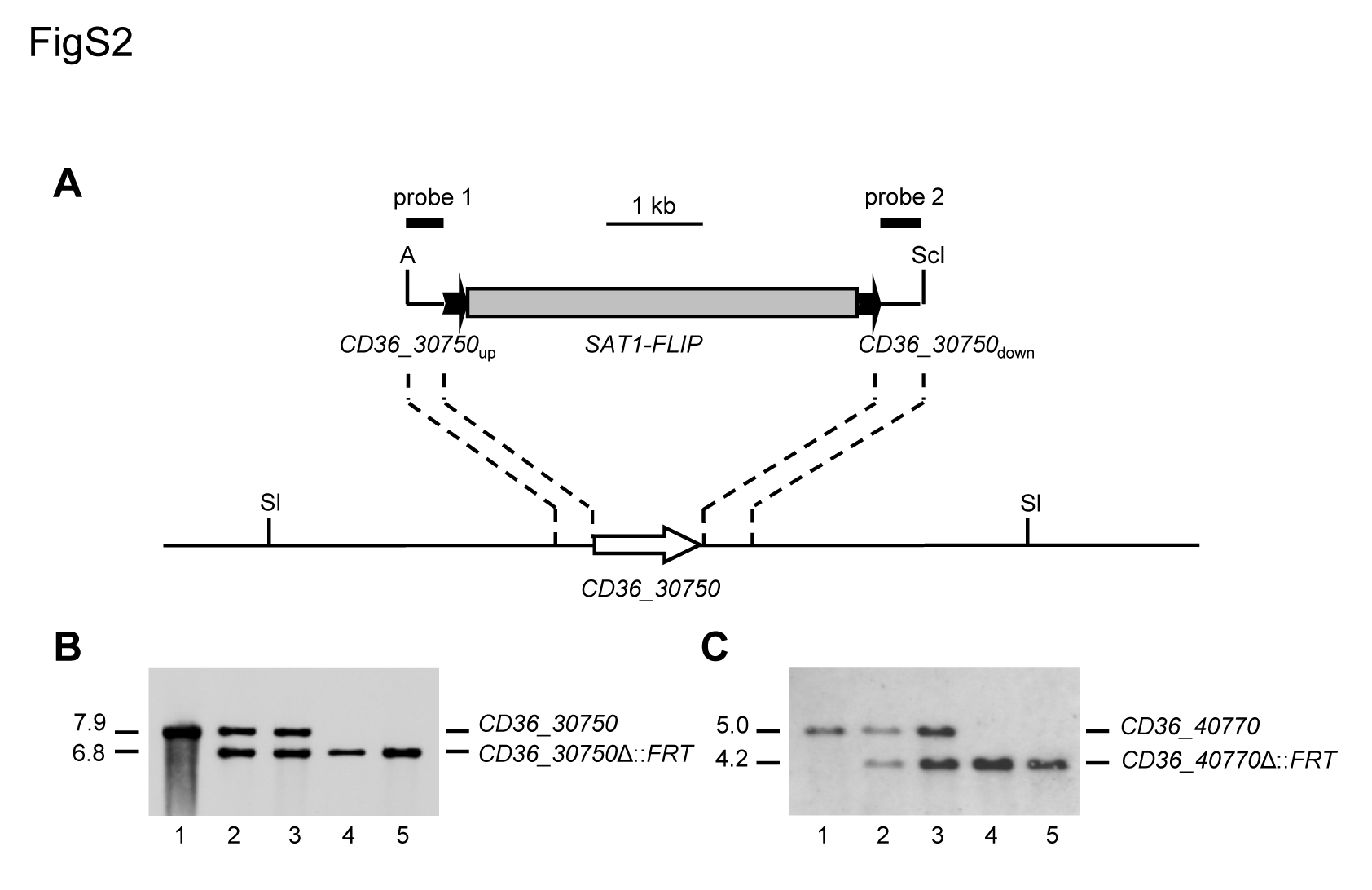

Supplement: Figure S2 — Construction of C. dubliniensis knock-out mutants in cdCSP1 ( CD36_30750 ) and cdCSP2 ( CD36_40770 ), respectively. (A) Structure of the deletion cassette from plasmid pCd30750M2 (top), which was used for deletion of both CD36_30750 alleles, and genomic structure of the CD36_30750 locus in strain Wü284 (bottom). The CD36_30750 coding region is represented by the white arrow, the upstream and downstream regions by the solid lines. The SAT1 flipper cassette is represented by a grey rectangle bordered by FRT sites (black arrows). The 34-bp FRT sites are not drawn to scale. The probes which were used for Southern analysis of the transformants are indicated by the black bars. Restriction sites used to cut out the linear fragment from the plasmid and for Southern analysis are given: A, ApaI; SI, SalI; ScI, SacI. (B) Southern hybridization of SalI-digested genomic DNA of parental strain Wü284 (lane 1), heterozygous CD36_30750Δ mutants Cd30750M2A (lane 2) and Cd30750M2B (lane 3), homozygous CD36_30750Δ mutants Cd30750M4A (lane 4) and Cd30750M4B (lane 5) with the CD36_30750-specific probe 1. The sizes of the hybridizing fragments (in kilobases) are given on the left side of the blot, and their identities on the right. (C) Southern hybridization of EcoRV-digested genomic DNA of parental strain Wü284 (lane 1), heterozygous CD36_40770Δ mutants Cd40770M2A (lane 2) and Cd40770M2B (lane 3), homozygous CD36_40770Δ mutants Cd40770M4A (lane 4) and Cd40770M4B (lane 5) with the CD36_40770-specific probe 1. The sizes of the hybridizing fragments are given on the left side of the blot, and their identities on the right. (TIF) [file pone.0061940.s002.tif]

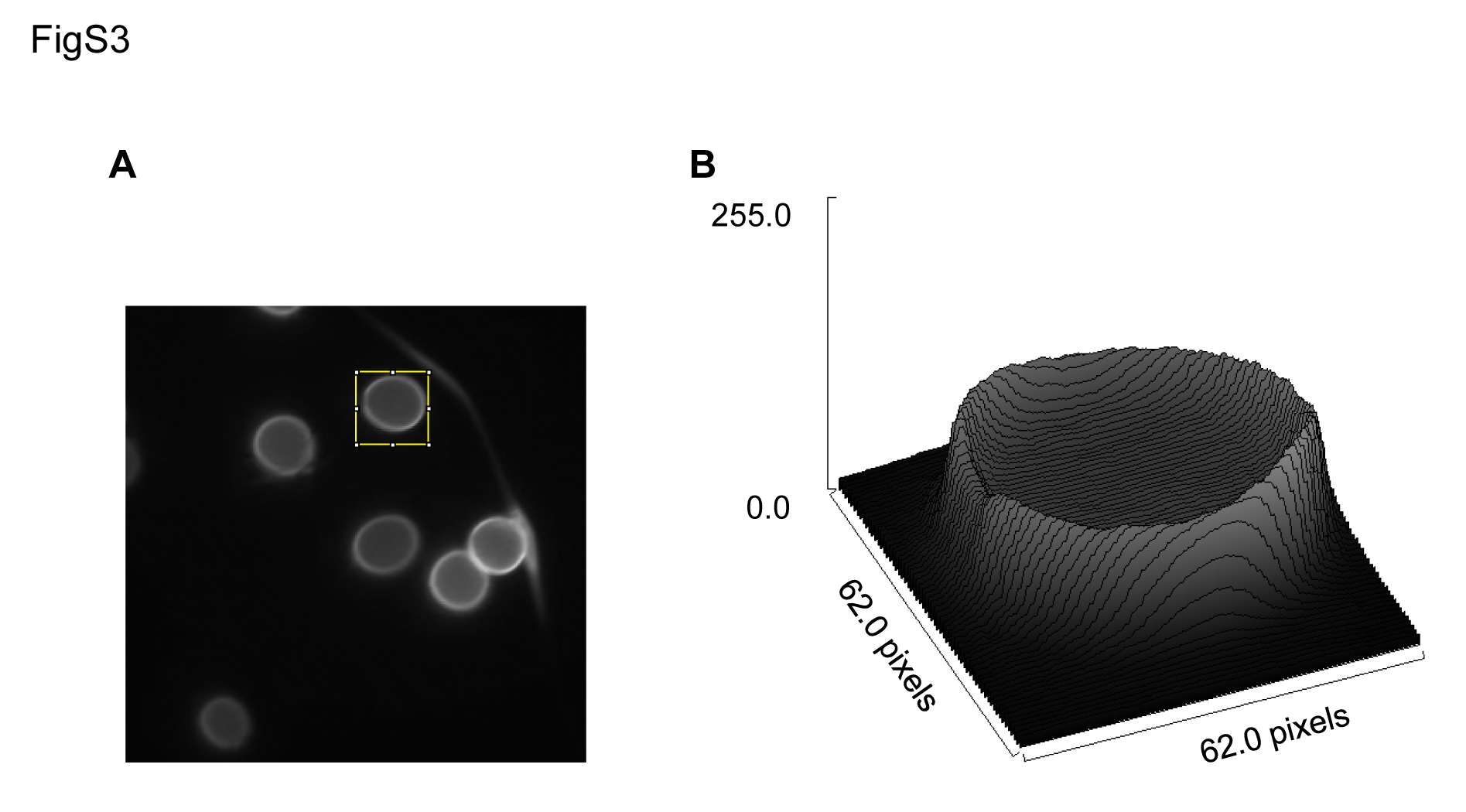

Supplement: Figure S3 — Protein localization of cdCsp1-GFP in chlamydospores. Fluorescence microscopy pictures of C. dubliniensis chlamydospores of strain Cd30750G1A after growth for 3 d at 25°C on rice-extract agar were analysed by surface plot analysis to localize the fluorescence signal of cdCsp1-GFP. (A) The yellow rectangle marks the area for the surface plot analysis. The intensity of fluorescence signal of cdCsp1-GFP within the defined region was determined by plot analysis (not shown) and surface plot analysis (B). The highest brightness was detected within the outer layer of the chlamydospore, suggesting that cdCsp1 is particularly located within the chlamydospore cell wall. (TIF) [file pone.0061940.s003.tif]

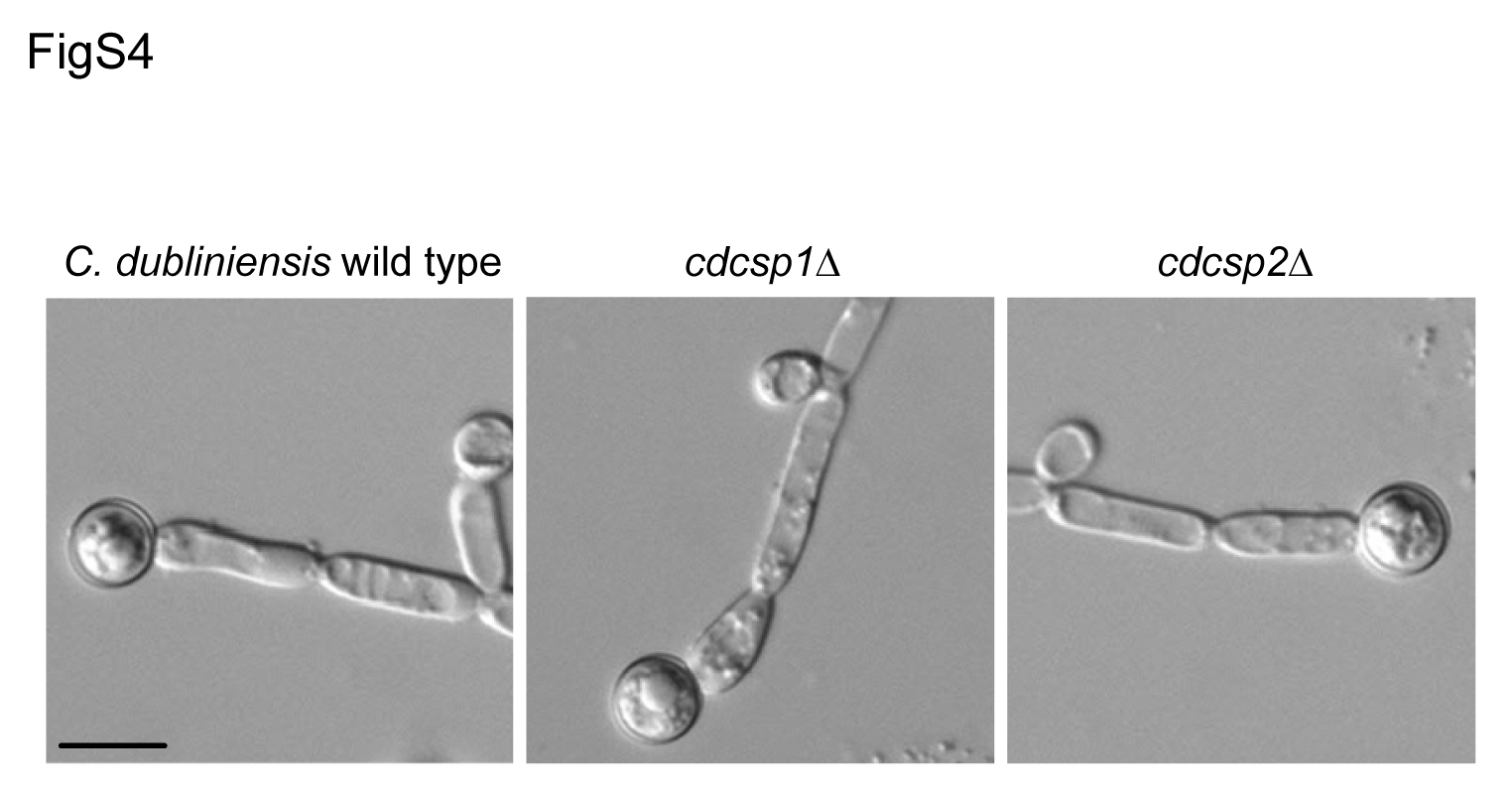

Supplement: Figure S4 — Phenotypic analysis of C. dubliniensis knock-out mutants in genes cdCSP1 and cdCSP2 , respectively. C. dubliniensis wild type Wü284 and mutant strains Cd30750M4A/B (cdcsp1Δ) and Cd40770M4A/B (cdcsp2Δ) were grown for 28 h in Staib medium, before cellular morphology was inspected by microscopy. Like the wild type control, both mutants efficiently produced chlamydospores under the tested conditions. For each mutant, the two independently constructed transformants (A/B) behaved identically and only one of them is shown. (TIF) [file pone.0061940.s004.tif]
