# Supplementary material for: Global Transcriptome Sequencing Identifies Chlamydospore Specific Markers in Candida albicans and Candida dubliniensis
Source: PLoS One. 2013 Apr 15;8(4):e61940. doi: 10.1371/journal.pone.0061940 (PMC3626690; doi:10.1371/journal.pone.0061940)
Supplement: Table S1 — Primers used in this study. (DOC) [file pone.0061940.s005.doc]

Table S1. Primers used in this study

| Primer^a^ | Sequence (5´-3´)^b^ |
| --- | --- |
| Ca3512-1 | GTT AGC TGA ATC CA**G** **GGC cC**T CAC TGT GTG G |
| Ca3512-2 | CCA GTC TTG AAA CAC **gga** **tcc** ATC ATG TTT TTG TTT C |
| Ca3512-3 | CGT GTT TCA AGA **CTG cag** TTG AAT AAC GCC AAC |
| Ca3512-4 | CAA AAT TAA TCA GCT AAC T**ga GCT C**GT TGC CTT AA |
| Ca3512-5 | GAC AAT TAT GAA CAT AAAC |
| Ca3512-6 | GCC ATG ACA AAT GC |
| Ca3512_fw1 | ACA CCA CTG CAA GTA TCC ATA TTG TGA |
| Ca3512_rev1 | ATC TTG TAT AAC CCT TTG TCG TCA AC |
| Ca4170-1 | GGT CCA ATA TAT C**Gg gCC** **C**GT GTT TCT TGA ATG G |
| Ca4170-2 | CCC ACT TAT TTG TGA CAA AC**g** **gat cc**A TCA TGT TGT TTC |
| Ca4170-3 | GTT AGT TTG TCA CA**c** **tgc** **AG**T GGG AGG GGG TTC |
| Ca4170-4 | GGT GAA CAT AAC AAT G**GA** **Gct c**CA ACT TAT C |
| Ca4170-5 | GTG AAA TCG ATA CTT ATG AAT GG |
| Ca4170-6 | CTC AAT CGT TCA TAC TGAAGG AAC |
| Ca4170_fw1 | GCT ACT GGT GAA ATT GTT GCT AAT C |
| Ca4170_rev1 | TCA TCA TCA CAG TCA TCG CTA TC |
| CD36_30750-1 | GGT GGG GTT C **gGG CCc** CCA TAT TCT ATT TTT AAG |
| CD36_30750-2 | GTT AAA GCA G **Ctc gAG** AAA ACT TCA TTT AAT G |
| CD36_30750-3 | GAT TAA ATC ATG TCT CAA **ccg cGG** TTT CGA ATA AC |
| CD36_30750-4 | ATC AGC TAA C **gaG CTC** GTT GGC TTA AAT CC |
| CD36_30750-5 | CCA ATC TTG AGA CAT **Gga TCC** ATC ATA CTT CTC TTT C |
| CD36_30750-6 | GAT TAA ATC ATG T**CT gcA G**AT GGT TTC GAA TAA C |
| CD36_30750-7 | GCA GTA GAA AAC TTC ATT **gtc gac** TAA GTT GAG TTG TTG |
| CD36_30750-8 | GTT ACT TAC AGT ACA GAG TTT GG |
| CD36_30750-9 | GCA AGG ATG ATG AAA AGT GCC ATC |
| CD36_30750_fw1 | CAA ATG CAA GGA TGA TGA AAA G |
| CD36_30750_rv1 | CTT CAG TGT GAA CAA TAG AGA A |
| CD36_40770-1 | GTT TAG ATA TAC A **ggG CCC** AGT ATA AAC TTC ATC |
| CD36_40770-2 | TGT AAA GCA G **CTc gAG** AAA ATT TCA TAT TGT G |
| CD36_40770-3 | CAG ATG AAA GAG **CCg Cgg** TTT CAG ATG ACA ATG |
| CD36_40770-4 | TAA AGC ATG GCC **gaG CTC** ACT TTG ATG AC |
| CD36_40770-5 | CTG GTT CTC ATA TGA CAA AG**g gat cc**A TCA TGT TGT TTC |
| CD36_40770-6 | CAA CAT GAT TAA TTG **CTg cag** CAT ATG AGA ACA CG |
| CD36_40770-7 | GGC GAT GAT GAC GAC AAT TCA AAA C |
| CD36_40770-8 | GCC TTA CAC AAG GAA GGA TTT TC |
| CD36_ACT1_fw1 | GAT GGT CAA GTT ATC ACT ATT GGT AAC GA |
| CD36_ACT1_rv2 | GAA CAA TAG ATG GAC CAG ATT CGT C |
| ACT1fw2 | TGG TAA CGA AAG ATT CAG AGC |
| ACT1rv2 | TCA TGA TGG AGT TGA AAG TGG |

**a.** Primers used for qRT-PCR are labeled with forward (fw) and reverse (rv), respectively.

**b.** Lowercase letters are nucleotide exchanges to create the bold restriction sites
